# Retraction Note: Neuropathological and neuroprotective features of vitamin B_12_ on the dorsal spinal ganglion of rats after the experimental crush of sciatic nerve: an experimental study

**DOI:** 10.1186/s13000-016-0578-z

**Published:** 2016-11-02

**Authors:** Rahim Hobbenaghi, Javad Javanbakht, Ehan Hosseini, Shahin Mohammadi, Mojtaba Rajabian, Pedram Moayeri, Mehdi Aghamohammad Hassan

**Affiliations:** 1 Department of Pathology, Faculty of Veterinary Medicine, University of Urmia, Urmia, Iran; 2 Department of Pathology, Faculty of Veterinary Medicine, University of Tehran, Tehran, Iran; 3 Faculty of Para Veterinary Medicine, Ilam University, Ilam, Iran; 4 Graduate Faculty of Veterinary Medicine, University of Urmia, Urmia, Iran; 5 Food Hygiene Department, University of Shahekord, Shahekord, Iran; 6 Resident of Large Animal Internal Medicine Department, University of Shahekord, Shahekord, Iran; 7 Department of Clinical Science, Faculty of Veterinary Medicine, University of Tehran, Tehran, Iran

## Retraction Note

The Editor-in-Chief and Publisher have retracted this article [1] because the scientific integrity of the content cannot be guaranteed. An investigation by the Publisher found it to be one of a group of articles we have identified as showing evidence suggestive of attempts to subvert the peer review and publication system to inappropriately obtain or allocate authorship. This article showed evidence of plagiarism (most notably from the articles cited [2–7]) and peer review and authorship manipulation.
